# Establishing a nomogram for post-hepatectomy liver failure prediction in hepatocellular carcinoma based on pre-operative and perioperative parameters

**DOI:** 10.3389/fmed.2025.1670396

**Published:** 2025-10-01

**Authors:** Danni Wang, Qingmei Ma, Lei Zhang, Jinxia Hou, Zhemei Zhang, Jing Wu, Jingheng Zhang

**Affiliations:** ^1^Department of Clinical Laboratory, Gansu Provincial Hospital, Lanzhou, Gansu, China; ^2^Gansu Provincial Medical Laboratory Clinical Medicine Research Center, Lanzhou, Gansu, China; ^3^Department of Outpatient, Gansu Provincial Hospital, Lanzhou, Gansu, China

**Keywords:** hepatocellular carcinoma (HCC), post-hepatectomy liver failure (PHLF), nomogram, pre-operative, perioperative

## Abstract

**Objective:**

Post-hepatectomy liver failure (PHLF) is a severe complication for hepatocellular carcinoma (HCC) patients post-surgery. This study explores PHLF risk factors and creates a nomogram for prediction using pre- and intraoperative factors.

**Methods:**

We retrospectively analyzed 654 patients who underwent hepatectomy. Eligible patients were randomly divided into training and internal validation cohorts in a 7:3 ratio. Key variables for nomogram construction were determined through integrated Least Absolute Shrinkage and Selection Operator (LASSO) and multivariate logistic regression analyses. The nomogram's performance was evaluated using receiver operating characteristic (ROC) curves, calibration curves, and decision curve analysis.

**Results:**

Among 228 eligible patients included in the study, 55 developed PHLF. Seven independent predictors were identified and incorporated into the nomogram: liver cirrhosis, total bilirubin (TBIL), prothrombin time (PT), Albumin-Bilirubin (ALBI), fibrosis-4 index (FIB4), ascites, and intraoperative blood loss. The nomogram demonstrated excellent predictive performance, with area under the curve (AUC) of 0.880 in the training cohort and 0.879 in the validation cohort. Calibration curve and decision curve analysis show that nomogram has significant clinical application value in predicting PHLF probability.

**Conclusion:**

We have developed and validated a novel PHLF risk prediction model that integrates pre-operative and intraoperative parameters, along with various liver function scoring systems, enabling more comprehensive and accurate prediction of PHLF risk in HCC patients.

## Introduction

Hepatocellular carcinoma (HCC) is the most common primary malignancy of the liver, with a rising global incidence posing a significant public health challenge (1). Notably, the prevalence of HCC exhibits marked geographic disparities, with high-incidence regions such as Asia, Africa, and South America. These variations are closely associated with the high prevalence of hepatitis B virus (HBV) infection in these regions (2, 3). Hepatectomy has long been recognized as a first-line treatment for HCC, offering substantial improvements in patient survival. Advances in surgical safety and post-operative outcomes have further enabled HCC patients to better combat this disease (4, 5). Particularly, the widespread adoption of laparoscopic techniques and the continuous refinement of surgical methods have solidified the role of curative hepatectomy in HCC management (6, 7). However, many HCC patients undergoing surgery often have pre-existing chronic conditions and compromised overall health, which pre-dispose them to post-operative complications such as bleeding, infection, bile leakage, wound infection, and post-hepatectomy liver failure (PHLF). PHLF is characterized as a clinical syndrome involving acute liver dysfunction after hepatectomy, manifested by jaundice, coagulopathy, and ascites (8). Its incidence varies significantly due to patient-specific factors, surgical expertise, and differences in perioperative management. The occurrence of PHLF severely impacts surgical outcomes and patient quality of life. Therefore, accurately identifying risk factors for PHLF is essential. Timely and precise pre-operative and intraoperative assessment of liver function and prediction of potential PHLF risks are crucial for its prevention and reducing post-operative mortality.

Compared with other patients, HCC patients are particularly prone to PHLF, and this phenomenon is especially obvious in HCC patients with underlying liver diseases, including hepatitis B, hepatitis C, alcoholic cirrhosis, non-alcoholic fatty liver disease, and autoimmune liver disease, etc. These patients have significantly reduced liver function reserve due to long-term damage to the liver parenchyma, insufficient remaining liver volume, or further deterioration of liver tissue quality after hepatectomy, thus greatly increasing the risk of PHLF (9, 10). It should be noted that there are also a small proportion of HCC patients in clinical practice whose liver parenchyma is normal, and the mechanism and risk characteristics of PHLF in these patients may differ from those of the former. Accurate assessment of pre-operative liver function reserve is critical for predicting PHLF. Over the years, several models, such as the Child-Pugh score, Model for End-Stage Liver Disease (MELD), and Albumin-Bilirubin (ALBI) grade, have been widely employed for pre-operative liver function evaluation. Additionally, indices like the Aspartate Aminotransferase-to-Platelet Ratio Index (APRI) and Fibrosis-4 (FIB-4) score have demonstrated notable performance in assessing significant fibrosis or cirrhosis (11–13). Although these models have been applied to predict PHLF with some success, there remains significant room for improvement in their predictive accuracy. To address this, integrating existing predictive models with additional pre-operative and intraoperative parameters for liver function assessment could provide a more comprehensive approach to predicting PHLF in HCC patients. Such models have significant clinical implications and research value for enhancing prediction precision and guiding perioperative management.

In this study, clinical and pathological data were retrospectively collected from HCC patients undergoing hepatectomy at Gansu Provincial People's Hospital. The study aimed to identify predictive factors associated with PHLF and develop a that incorporates pre-operative and intraoperative clinical and pathological factors to predict PHLF risk in HCC patients. This approach aims to optimize pre-operative treatment strategies, reduce the incidence of PHLF, and enhance the safety and efficacy of HCC treatment.

## Materials and methods

This study analyzed data from HCC patients who underwent partial hepatectomy at Gansu Provincial Hospital between 2020 and 2023. Initially, 645 patients who underwent hepatectomy for hepatocellular carcinoma were screened; after screening according to the inclusion and exclusion criteria, 228 patients who underwent HCC resection were finally included. Patients were divided into PHLF and non-PHLF groups based on post-operative liver failure occurrence. This study was approved by the Medical Ethics Committee of Gansu Provincial Hospital (ID: 2024-817). We confirm that all methods and procedures were performed in accordance with relevant guidelines and regulations. Inclusion criteria were: (1) pathologically confirmed HCC patients; (2) first-time liver cancer resection surgery; (3) pre-operative assessment showing adequate cardiopulmonary function for surgery. Exclusion criteria were: (1) patients who have previously undergone liver resection, HCC-related interventions (such as ablation, embolization, etc.) or targeted drug therapy; (2) insufficient pre-operative laboratory or pathological data; (3) patients with other malignancies, severe cardiovascular disease, renal insufficiency, or severe encephalopathy; and (4) patients requiring vascular reconstruction during hepatectomy. PHLF was diagnosed according to the International Study Group of Liver Surgery definition and diagnostic criteria. PHLF was defined as abnormal serum bilirubin levels and International Normalized Ratio (INR) on or after post-operative day 5. Elevated INR and hyperbilirubinemia were defined according to local laboratory normal threshold ranges. Based on laboratory definitions, post-operative total bilirubin >34.2 mmol/L was defined as hyperbilirubinemia, and INR >1.30 was defined as elevated INR.

Multiple clinical parameters were collected through the hospital's clinical record system during hospitalization, including: age, gender, pre-operative BMI, smoking history, drinking history, diabetes history, hepatitis B infection history, cirrhosis history, pre-operative laboratory tests (including complete blood count, liver function, renal function, and alpha-fetoprotein), total bilirubin (TBIL), prothrombin time (PT), ALBI score, FIB4 score, tumor size, tumor number, intraoperative blood loss, and post-operative liver failure occurrence. FIB-4, aspartate aminotransferase-to-neutrophil ratio (ANRI), aspartate aminotransferase-to-lymphocyte ratio (ALRI), platelet-to-lymphocyte ratio (PLR), ALBI, and FIB-4 scores were calculated (14). Univariate and multivariate logistic regression analyses were performed on these clinical pathological indicators to screen for independent risk factors affecting PHLF occurrence.

### Ethical approval

This study was approved by the Medical Ethics Committee of Gansu Provincial Hospital (ID: 2024-817). Written consent was obtained from the study participants, All participants were provided informed consent during the study. The confidentiality of the participants was assured by anonymizing personal identifiers in the data. All methods and procedures in the study were performed in accordance with the Declaration of Helsinki.

### Statistical methods

**Figure 1:**
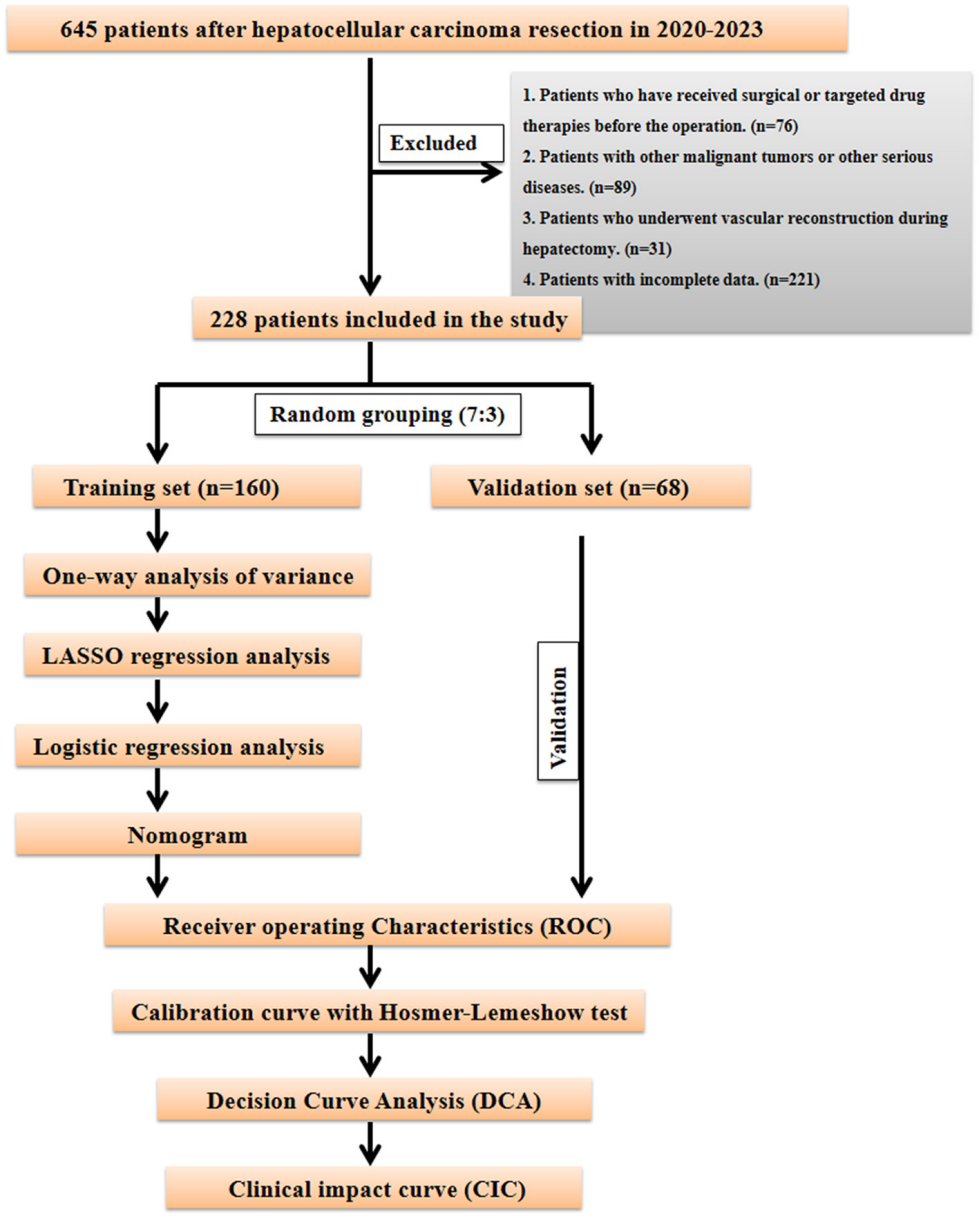
Flowchart of the research process.

Data analysis was performed using SPSS 23.0 statistical software, with univariate and multivariate analyses conducted at α = 0.05 level. Chi-square tests were used for comparison between groups for categorical variables. To maintain balance between training and internal validation sets, R 3.3.2 software was used to randomly divide the multicenter data in a 7:3 ratio. In the training cohort, predictive factors for PHLF formation were first screened through univariate analysis, then optimal predictive variables were selected through the Least Absolute Shrinkage and Selection Operator (LASSO) regression model. Based on the selected variables, risk factors were determined through multivariate Logistic regression analysis to establish the prediction model. To comprehensively evaluate the model's performance and clinical application value, we constructed a nomogram model and generated receiver operating characteristic (ROC) curves, calibration curves, decision curve analysis (DCA), and clinical impact curves (CIC). Calibration curves play a crucial role in evaluating the accuracy of disease risk prediction models, precisely measuring the consistency between predicted risks and actual outcome events. Good calibration indicates reliable prediction results, while poor calibration suggests the model may overestimate or underestimate disease risk. Decision curve analysis (DCA) primarily evaluates the model's ability to support clinical decision-making, demonstrating predictive outcomes for interventions or treatment strategies at different decision thresholds. The clinical impact curve (CIC) visually presents the proportion of benefits and losses at different probability thresholds. Through these analytical methods, this study aims to comprehensively and thoroughly evaluate the constructed model's performance and clinical utility in predicting PHLF, providing robust decision support for clinical practice. The specific research flow is detailed in Figure 1.

## Results

### Clinical characteristics of patients

The study included 228 patients randomly divided into training and internal validation sets in a 7:3 ratio (Figure 1). Among them, 55 patients developed post-operative liver failure, with 40 cases in the training set and 15 in the validation set (Supplementary Table S1). Statistical analysis showed no significant differences in factors between the training and validation sets (*P* > 0.05). The patients' baseline characteristics are shown in Supplementary Table S2.

### Identification of predictive factors

**Figure 2:**
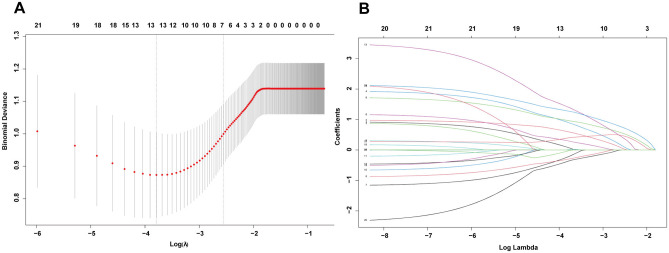
**(A)** The optimal parameter (λ) selection in the LASSO model employed fivefold cross-validation using a minimum criteria approach. The optimal values of λ are represented by dotted vertical lines. **(B)** LASSO coefficient profiles of seven clinical features. The plot was created using a logarithmic scale for the lambda values. A vertical line was added to indicate the lambda value selected through fivefold cross-validation.

Given the numerous variables involved, in the training set, we first used the LASSO regression algorithm to further screen 21 statistically significant predictive factors identified through univariate analysis from Supplementary Table S1. Seven candidate predictive factors were identified: liver cirrhosis, total bilirubin (TBIL), prothrombin time (PT), albumin-bilirubin grade (ALBI), fibrosis-4 index (FIB4), ascites, and intraoperative blood loss, as detailed in Figures 2A, B. Subsequently, for these seven characteristic predictive factors, we performed logistic multivariate regression analysis using the “rms” package in R program, employing multivariate stepwise logistic regression to identify independent predictive factors and construct the prediction model. The analysis revealed that liver cirrhosis, TBIL, PT, ALBI, FIB4, ascites, and intraoperative blood loss were all independent predictors of PHLF in HCC patients. Detailed results of the multivariate analysis are shown in Table 1.

**Table 1:** Multivariable analysis in the training cohort.

**Variables**	**β**	**OR (95%CI)**	***P-*value**
Intercept	−4.388	0.012 (0.003–0.050)	<0.001
**Liver cirrhosis**			
No			
Yes	1.204	3.333 (1.155–9.620)	0.026
**TBIL (**μ**mol/L)**			
≤ 34.2			
>34.2	1.345	3.840 (1.028–14.342)	0.045
**PT(s)**			
≤ 14			
>14	1.525	4.594 (1.641–12.863)	0.004
**ALBI**			
≤ -2.39			
>-2.39	2.288	9.857 (1.171–83.008)	0.035
**FIB4**			
≤ 2.67			
>2.67	1.060	2.886 (1.030–8.083)	0.044
**Ascites**			
No			
Yes	1.714	5.550 (1.823–16.897)	0.003
Intraoperative blood loss (ml)	0.001	1.001 (1.001–1.002)	0.002

OR, odds ratio; CI, confidence interval; TBIL, total bilirubin; PT, prothrombin time; ALBI, albumin bilirubin score; FIB-4, fibrosis index.

### Prediction model construction

**Figure 3:**
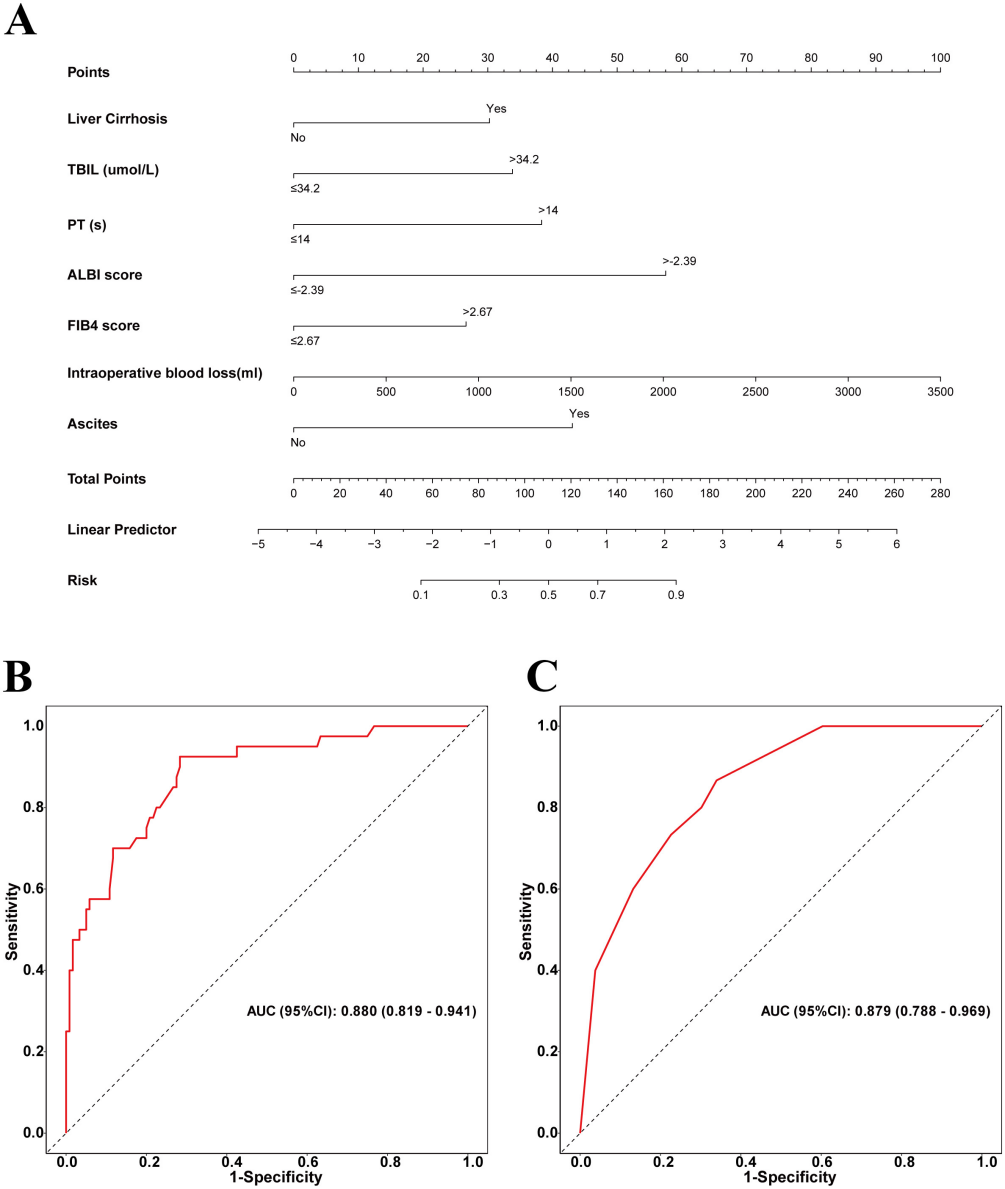
**(A)** Nomogram with FBI4, TBIL, ALBI, Liver Cirrhosis, Ascites, Intraoperative blood loss, and PT procedures predicts the PHLF. **(B)** The area under the AUC for the discrimination of the model in the training set, AUC (95% CI): 0. 880 (0.819–0.941); **(C)** the area under the AUC for the discrimination of the model in the validation set, AUC (95% CI): 0.879 (0.788–0.969).

By integrating these core clinical features, we constructed a PHLF prediction model based on the above seven independent predictors and developed a nomogram. The nomogram clearly reveals the relative weight of each factor in PHLF risk occurrence. According to the nomogram, intraoperative blood loss (>2,000 ml) is the strongest predictor, followed by ALBI (>-2.39), ascites, PT (>14 s), TBIL (>34.2 μmol/L), presence of liver cirrhosis, and FIB4 score (>2.67; Figure 3A).

### Prediction model performance evaluation

For this sample, in the training set, the nomogram's area under the curve (AUC) for predicting PHLF was 0.880 (95% confidence interval: 0.819–0.941), while in the validation set, the AUC remained high at 0.879 (95% confidence interval: 0.788–0.969), further demonstrating the model's strong discriminative ability (Figures 3B, C).

### Calibration curves of the prediction model

**Figure 4:**
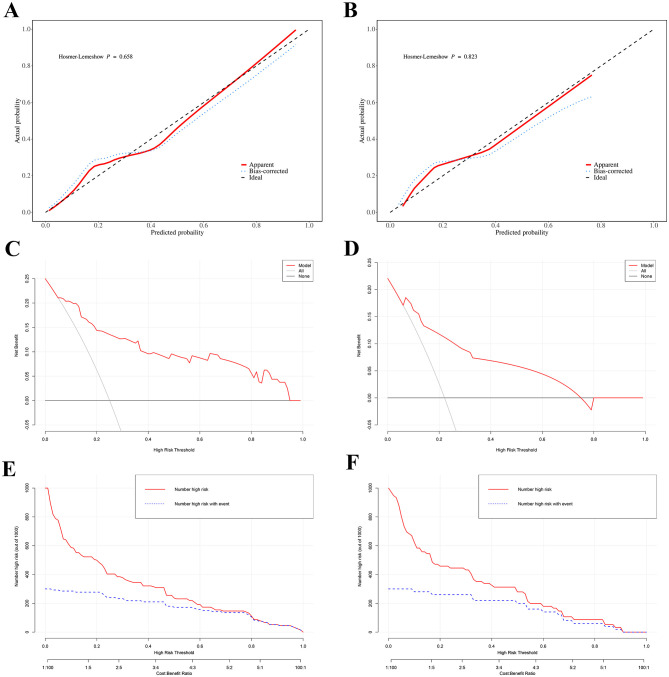
Calibration curves of the predicted probabilities of post-operative PHLF in the training cohort **(A)** and the validation cohort **(B)**, all *P* > 0.05 in the Hosmer–Lemeshow test suggested an agreement between the predicted probabilities and observed outcomes; Decision curve analysis (DCA) for post-operative PHLF nomogram. The black line represents the assumption that no patients develop PHLF, while the gray line assumes that all patients develop PHLF. The blue line corresponds to the risk prediction model. This analysis was conducted in both the training set **(C)** and the validation set **(D)**; Clinical impact curve (CIC). The red curve (number of high-risk individuals) indicates the number of people who are classified as positive (high risk) by the model at each threshold probability; the blue curve (number of high-risk individuals with outcome) is the number of true positives at each threshold probability. CIC visually indicated that nomogram conferred high clinical net benefit and it confirmed the clinical value of the nomogram in PHLF. This analysis was conducted in both the training set **(E)** and the validation set **(F)**.

Additionally, calibration curves showed no differences between predicted and actual probabilities of post-operative liver failure occurrence in both the training set (Hosmer–Lemeshow *P* = 0.658) and validation set (Hosmer–Lemeshow *P* = 0.823; Figures 4A, B). The results show that the prediction model's calibration curve fits well with the standard curve, indicating good calibration of the predictive model and good consistency between predicted and actual values, demonstrating excellent model performance.

### Clinical application

Furthermore, decision curve analysis (DCA) results indicate that the nomogram can bring significant net benefits to patients with post-operative liver failure (Figures 4C, D). The *x*-axis represents the threshold probability, and the *y*-axis represents net benefit. The black horizontal line indicates no PHLF occurrence in all patients, with net benefit of 0; the gray diagonal line indicates PHLF occurrence in all patients. The DCA of this study cohort (red solid line) indicates that using this nomogram to predict PHLF is more beneficial for patient prognosis compared to assuming all or no patients develop PHLF. The clinical impact curve (CIC) analysis, shown in Figures 4E, F, was designed to assess the clinical applicability of the risk prediction graph. The CIC intuitively demonstrates that the nomogram has superior overall net benefit and impacts patient outcomes across a wide range of practical threshold probabilities, showing significant clinical utility in predicting PHLF probability in patients.

## Discussion

Hepatocellular carcinoma (HCC), as one of the most common malignant tumors globally, shows significant variations in incidence and mortality rates influenced by factors such as gender, age, and etiology. Although treatment options for HCC are diverse, including radiotherapy, targeted therapy, and chemotherapy, radical hepatectomy remains the preferred treatment option for HCC patients (15–17). It is worth noting that in certain clinical scenarios, liver transplantation, as a treatment method with the dual value of radical tumor treatment and liver function replacement, demonstrates superior oncological efficacy and has become a more favored option for some patients. However, it is noteworthy that some patients often have concurrent chronic liver diseases pre-operatively, with cirrhosis being particularly common, which significantly increases the risk of post-hepatectomy liver failure (PHLF). Therefore, PHLF prevention holds paramount importance in clinical practice. The ability to accurately predict PHLF and implement appropriate interventions, especially immediately before or after surgery, is crucial for treatment decision-making and improving post-operative prognosis in hepatectomy patients. Current research shows that various clinical comprehensive scoring systems have certain value in PHLF prediction but also have limitations, with most restricted to pre-operative prediction. Thus, exploring more comprehensive and accurate pre-operative assessment methods to improve the prediction accuracy of PHLF risk and implement timely interventional treatment has become a key measure for improving patients' post-operative survival (12, 18). In this study, we constructed an innovative PHLF prediction model that organically integrates pre-operative and intraoperative indicators along with various liver function clinical scoring systems. Specifically, the included pre-operative predictors comprise liver cirrhosis, total bilirubin (TBIL), prothrombin time (PT), and ascites; the intraoperative predictor is intraoperative blood loss; and clinical scoring systems include albumin-bilirubin grade (ALBI) and fibrosis-4 index (FIB4). We hope that through this comprehensive prediction model, clinicians will have a more effective tool to prevent and address PHLF occurrence.

Previous studies have indicated that cirrhosis is an extremely significant factor affecting post-operative complications (19–21). In cirrhotic conditions, a large number of hepatocytes are replaced by fibrous tissue, leading to a sharp reduction in normal hepatocyte count. Hepatectomy surgery causes further damage to the liver, making it difficult for remaining hepatocytes to handle the body's metabolic and detoxification functions. Additionally, liver blood flow conditions change. The surgical process may affect liver blood perfusion, and cirrhotic patients' hepatic vascular systems are already disordered. Surgery further reduces liver blood and oxygen supply, and their liver regeneration rate is slower compared to non-cirrhotic patients. Hepatocytes suffer damage due to insufficient nutrition and oxygen supply, undoubtedly increasing the risk of post-hepatectomy liver failure (PHLF). In our study, both cirrhosis and FIB4 score were confirmed as independent risk factors for PHLF, with patients having higher FIB4 scores being more susceptible to PHLF, further indicating that PHLF risk increases with the progression of liver fibrosis and cirrhosis. Prothrombin time (PT) and total bilirubin (TBIL) were also incorporated into the prediction model; as key indicators for assessing liver functional reserve, they play crucial roles in predicting mortality risk from PHLF. These two indicators reflect liver function from different dimensions—prothrombin time levels reflect the liver's ability to synthesize coagulation factors, while total bilirubin levels reflect the liver's glucuronidation function and ability to secrete bilirubin into bile ducts. After hepatectomy, the liver must undertake the remaining arduous tasks of metabolism and synthesis. When both indicators reach abnormal levels by post-operative day 5, it suggests severe liver dysfunction, indicating that remaining hepatocytes cannot effectively compensate, predicting poor prognosis. Numerous clinical studies have confirmed (12, 22, 23) that prothrombin time and total bilirubin have high accuracy and reliability in predicting adverse outcomes in PHLF patients, helping doctors identify high-risk PHLF patients promptly for more aggressive treatment measures and enhanced monitoring.

The ALBI score is a simple model based on pre-operative serum albumin and bilirubin levels for assessing liver functional reserve. This scoring method is simple to operate and highly accessible, requiring only two objective indicators—total bilirubin and albumin—for calculation, and these two observational indicators are independent and unaffected by subjective factors. Multiple studies have shown (21, 24–26) that compared to Child-Pugh and MELD scores, the ALBI score more accurately reflects liver functional reserve status and has greater advantages in predicting PHLF occurrence and prognosis in liver cancer patients. Additionally, Yi-Bo Tian et al. (11) found that the combined ALBI-FIB4 score showed excellent performance in predicting PHLF, severe PHLF, and post-operative mortality in liver cancer hepatectomy patients, superior to currently used liver function and fibrosis scoring systems. Through multivariate regression analysis, our study identified both ALBI score and FIB-4 index as independent predictors of PHLF. This combination allows simultaneous observation of liver function and fibrosis status, making it feasible to improve PHLF prediction accuracy. Furthermore, ascites formation is closely related to liver function, which is one reason for its inclusion in the nomogram.

In liver disease research, accurate prediction of PHLF risk in hepatocellular carcinoma (HCC) patients after surgery remains a core clinical focus. Notably, our study found that massive intraoperative blood loss significantly increases patients' risk of developing PHLF. When massive blood loss occurs during surgery, effective circulating blood volume decreases immediately, leading to insufficient liver perfusion. Normal liver function highly depends on adequate blood supply to deliver oxygen and nutrients; when perfusion is insufficient, hepatocytes become damaged due to lack of oxygen and essential nutrients. Moreover, during the process of restoring perfusion through blood transfusion and fluid replacement after intraoperative bleeding, ischemia-reperfusion injury occurs, which can directly cause hepatocyte damage or death. This mechanism plays a key role in the pathophysiological process of post-operative liver failure (27). Previous studies have clearly shown (20, 28) that intraoperative blood loss >400 ml is a risk factor for PHLF development. Based on in-depth analysis of intraoperative and pre-operative factors, we constructed an innovative prediction model that not only incorporates predictive indicators related to liver functional reserve and liver fibrosis but also fully considers the key factor that massive intraoperative blood loss increases patients' post-operative PHLF risk. A limitation of this study is the lack of complete intraoperative blood transfusion records. Although estimated intraoperative blood loss offers some reference, actual transfusion status more objectively reflects circulatory state, bleeding severity, and clinical interventions, thus representing a more valuable clinical indicator.

Specifically, this model integrates multiple independent predictors from both intraoperative and pre-operative phases, including ALBI score, FIB-4 index, ascites, cirrhosis, prothrombin time, total bilirubin, and intraoperative blood loss. When compared to established models such as Child-Pugh and MELD, our model exhibits distinct advantages. The Child-Pugh score, while widely used for assessing liver function, primarily focuses on post-operative liver reserve with parameters like bilirubin, albumin, and ascites, lacking consideration of intraoperative factors such as blood loss that are critical for post-hepatectomy liver failure (PHLF) in HCC patients. MELD, on the other hand, is more oriented toward predicting short-term mortality in patients with end-stage liver disease and does not specifically target the unique pathophysiological changes and surgical risks associated with HCC resection. In contrast, our model offers more comprehensive consideration dimensions by incorporating both pre-operative liver function indicators (ALBI score, FIB-4 index, cirrhosis), and intraoperative variables (blood loss). This integration allows for a more targeted assessment of PHLF risk in the context of HCC resection, providing higher reliability and comprehensiveness in prediction. In terms of model performance evaluation, comparison of area under the curve (AUC) values revealed that our model demonstrates excellent discriminative ability in accurately distinguishing patients likely to develop post-operative PHLF from others. Additionally, calibration curve and decision curve analysis results strongly confirm that the nomogram based on this model shows excellent performance in predicting PHLF risk, demonstrating good predictive effects. This fully indicates that this model can provide extremely valuable reference information for clinical risk monitoring and physician decision-making processes. To effectively integrate the nomogram into clinical practice, the following measures can be taken: develop user-friendly digital tools or mobile applications that can automatically calculate the PHLF risk by inputting parameters and visualize the risk stratification, reducing human errors and saving time; formulate standard checklists consistent with key predictive indicators such as ALBI classification, FIB4 index, ascites, liver cirrhosis and intraoperative blood loss, ensuring the consistency of data collection for pre-operative assessment and intraoperative records, and promoting teamwork. This model has significant practical value in clinical settings, helping doctors efficiently identify HCC patients with high post-operative PHLF risk, and providing strong support for personalized care and management.

However, limited by data availability, both model development and validation were completed internally. While this approach allowed us to fully optimize the model in a relatively controlled environment (29), it also somewhat limits the model's generalizability to other institutions. As is well-known, external validation can provide more rigorous assessment of model robustness, and biases present in external validation datasets might potentially mask true model performance discovered through internal validation. Furthermore, since this study was conducted only among patients from a single medical institution, completely eliminating selection and information biases associated with single sampling remains challenging. Therefore, conducting multicenter and prospective cohort studies becomes particularly necessary for more in-depth exploration of this model's performance and application range. Moving forward, we plan to address this limitation through multi-center studies validating our findings in diverse cohorts to confirm the nomogram's reliability, enhance its clinical applicability, and support decision-making in preventing and managing post-hepatectomy liver failure across broader clinical settings.

In conclusion, thoroughly understanding risk factors for PHLF in HCC patients before and during surgery is of great significance for reasonable treatment plan selection and post-operative prognosis. In this study, we successfully developed and validated a nomogram model for predicting post-operative liver failure, which showed significant effectiveness in predicting PHLF occurrence in HCC patients undergoing hepatectomy. With this model, clinicians can efficiently identify HCC patients at higher risk for post-operative PHLF, thereby providing strong support for implementing personalized patient care and management, helping to improve clinical treatment effects and patient prognosis quality.

## Data Availability

The original contributions presented in the study are included in the article/Supplementary material, further inquiries can be directed to the corresponding author.
