# Supplementary material for: Establishing a nomogram for post-hepatectomy liver failure prediction in hepatocellular carcinoma based on pre-operative and perioperative parameters
Source: Front Med (Lausanne). 2025 Oct 1;12:1670396. doi: 10.3389/fmed.2025.1670396 (PMC12521178; doi:10.3389/fmed.2025.1670396)
Supplement: Supplementary file 1 [file Table_1.docx]

Table S1 Baseline characteristics between the PHLF and non-PHLF groups

| Characteristic |  | PHLF | | | *P* |
| --- | --- | --- | --- | --- | --- |
|  |  | No (n= 173)  N (%) | | Yes (n= 55)  N (%) |  |
| Age |  |  | |  | 0.191 |
|  | ≤50 | 43(24.9) | | 9(16.4) |  |
|  | >50 | 130(75.1) | | 46(83.6) |  |
| Sex |  |  | |  | 0.253 |
|  | Male | 125(72.3) | | 44(80.0) |  |
|  | Female | 48(27.7) | | 11(25.9) |  |
| BMI(kg/m²) |  |  | |  | 0.095 |
|  | ≤24 | 113(65.3) | | 43(78.2) |  |
|  | >24 | 60(34.7) | | 12(21.8) |  |
| Hepatitis B virus infection |  |  | |  | 0.499 |
|  | No | 75(43.4) | | 21(38.2) |  |
|  | Yes | 98(56.6) | | 34(61.8) |  |
| Smoking |  |  | |  | 0.074 |
|  | No | 142(82.1) | | 39(70.9) |  |
|  | Yes | 31(17.9) | | 16(29.1) |  |
| Drinking |  |  | |  | 0.022 |
|  | No | | 149(86.1) | 40(72.7) |  |
|  | Yes | | 24(13.9) | 15(27.3) |  |
| Hypertensive |  | |  |  | 0.880 |
|  | No | | 140(80.9) | 44(80.8) |  |
|  | Yes | | 33(19.1) | 11(20.0) |  |
| Diabetes |  | |  |  | 0.986 |
|  | No | | 148(85.5) | 47(85.5) |  |
|  | Yes | | 25(14.5) | 8(14.5) |  |
| Liver Cirrhosis |  | |  |  | <0.001 |
|  | No | | 96(55.5) | 13(23.6) |  |
|  | Yes | | 77(44.5) | 42(76.4) |  |
| HBsAg |  | |  |  | 0.308 |
|  | No | | 60(34.7) | 15(27.3) |  |
|  | Yes | | 113(65.3) | 40(72.7) |  |
| AFP(ng/mL) |  | |  |  | 0.979 |
|  | ≤400 | | 123(71.1) | 39(70.9) |  |
|  | >400 | | 50(28.9) | 16(29.1) |  |
| CEA(ng/mL) |  | |  |  | 0.220 |
|  | ≤5 | | 145(84.3) | 50(90.9) |  |
|  | >5 | | 27(15.7) | 5(9.1) |  |
| ALB(g/L) |  | |  |  | <0.001 |
|  | ≤35 | | 25(14.5) | 20(36.4) |  |
|  | >35 | | 148(85.5) | 35(63.6) |  |
| TBIL (umol/L) |  | |  |  | 0.008 |
|  | ≤34.2 | | 156(90.2) | 42(76.4) |  |
|  | >34.2 | | 17(9.8) | 13(23.6) |  |
| ALP(U/L) |  | |  |  | 0.030 |
|  | ≤135 | | 130(75.1) | 33(60.0) |  |
|  | >34 | | 43(24.9) | 22(40.0) |  |
| ALT (u/L) |  | |  |  | 0.150 |
|  | ≤40 | | 104(60.1) | 27(49.1) |  |
|  | >40 | | 69(39.9) | 28(50.9) |  |
| AST (u/L) |  | |  |  | <0.001 |
|  | ≤35 | | 91(52.6) | 13(23.6) |  |
|  | >35 | | 82(47.4) | 42(76.4) |  |
| HB(g/L) |  | |  |  | 0.019 |
|  | ≤120 | | 19(11.0) | 13(23.6) |  |
|  | >120 | | 154(89.0) | 42(76.4) |  |
| PLT (10^9^/L) |  | |  |  | <0.001 |
|  | ≤100 | | 33(19.1) | 26(47.3) |  |
|  | >100 | | 140(80.9) | 29(52.7) |  |
| PT(s) |  | |  |  | <0.001 |
|  | ≤14 | | 110(63.6) | 16(29.1) |  |
|  | >14 | | 63(63.4) | 39(70.9) |  |
| INR |  | |  |  | 0.025 |
|  | ≤1.3 | | 169(97.7) | 50(90.9) |  |
|  | >1.3 | | 4(2.3) | 5(9.1) |  |
| ALBI |  | |  |  | <0.001 |
|  | ≤-2.6 | | 100(57.8) | 16(29.1) |  |
|  | -2.6- -2.39 | | 70(40.5) | 32(58.2) |  |
|  | >-2.39 | | 3(1.7) | 7(12.7) |  |
| PALBI |  | |  |  | 0.008 |
|  | ≤-2.27 | | 122(70.5) | 28(50.9) |  |
|  | >-2.27 | | 51(29.5) | 27(19.1) |  |
| APRI |  | |  |  | <0.001 |
|  | ≤0.5 | | 62(35.8) | 9(16.4) |  |
|  | 0.5-1.5 | | 82(47.4) | 20(36.4) |  |
|  | 1.5-2.5 | | 18(10.4) | 14(25.5) |  |
|  | >2.5 | | 11(6.4) | 12(21.8) |  |
| ANRI |  | |  |  | 0.234 |
|  | ≤30 | | 160(92.5) | 48(87.3) |  |
|  | >30 | | 13(7.5) | 7(12.7) |  |
| PLR |  | |  |  | 0.017 |
|  | ≤100 | | 63(36.4) | 30(54.5) |  |
|  | >100 | | 110(63.6) | 25(45.5) |  |
| FIB4 |  | |  |  | <0.001 |
|  | ≤1.30 | | 88(50.9) | 11(20.0) |  |
|  | 1.30-2.67 | | 55(31.8) | 14(30.3) |  |
|  | >2.67 | | 30(17.3) | 30(54.5) |  |
| Intraoperative blood loss (ml) |  | | 408.70±493.21 | 610.54±781.76 | 0.024 |
| Number of tumors |  | | 1.49±0.50 | 1.49±0.51 | 0.945 |
| Ascites |  | |  |  | <0.001 |
|  | No | | 153(88.4) | 29(52.7) |  |
|  | Yes | | 20(11.6) | 26(47.3) |  |
| Tumor diameter(cm) |  | |  |  | 0.945 |
|  | <5 | | 89(51.4) | 28(50.9) |  |
|  | ≥5 | | 84(48.6) | 27(49.1) |  |
| Tumor differentiation type |  | |  |  | 0.010 |
|  | Highly - moderately | | 113(65.3) | 46(83.6) |  |
|  | Poorly | | 60(34.7) | 9(16.4) |  |
| Tumor thrombus |  | |  |  | 0.961 |
|  | No | | 134(77.5) | 42(77.8) |  |
|  | Yes | | 39(22.5) | 12(22.2) |  |

Note: BMI, body mass index ;AFP, alpha-fetoprotein; CEA, Carcinoembryonic antigen; ALB, albumin; TBIL, total bilirubin; ALP, Alkaline phosphatase ; AST, aspartate transaminase; ALT, alanine transaminase; GGT, glutamyltranspeptidase; HB , hemoglobin; PLT, platelet count; PT, prothrombin time; INR, International normalized ratio; ALBI, albumin bilirubin score; PALBI, Platelet - Albumin - Bilirubin Index, APRI, aspartate transaminase to Platelet Ratio ; ANRI, aspartate aminotransferase-neutrophil ratio; PLR, platelet-to-lymphocyte ratio; FIB-4, fibrosis index;HBsAg, hepatitis B surface antigen;

Table S2 Baseline characteristics of participants in training and validation set

| Variables |  | | Total | | Training cohort | Validation cohort | P |
| --- | --- | --- | --- | --- | --- | --- | --- |
|  |  | | (n= 228) | | n=160 | n=68 |  |
| Age |  | |  | |  |  | 0.865 |
|  | ≤50 | | 52（22.8） | | 36（22.5） | 16（23.5） |  |
|  | >50 | | 176（77.2） | | 124（77.5） | 52（76.5） |  |
| Sex |  | |  | |  |  | 0.261 |
|  | Male | | 169（71.1） | | 122（76.3） | 47（69.1） |  |
|  | Female | | 59（25.9） | | 38（23.7） | 59（25.9） |  |
| BMI(kg/m²) |  | |  | |  |  | 0.431 |
|  | ≤24 | | 156（68.4） | | 112（70.0） | 44（64.7） |  |
|  | >24 | | 72（31.6） | | 48（30.0） | 24（35.3） |  |
| Hepatitis B virus infection |  | |  | |  |  | 0.466 |
|  | No | | 75（32.9） | | 55（34.4） | 20（29.4） |  |
|  | Yes | | 153（67.1） | | 105（65.6） | 48（70.6） |  |
| Smoking |  | |  | |  |  | 0.466 |
|  | No | | 182(79.8) | | 126(78.8) | 56(82.4) |  |
|  | Yes | | 46(20.2) | | 34(21.2) | 12(24.6) |  |
| Drinking |  | |  | |  |  | 0.796 |
|  | No | 190(83.3) | | 134(83.8) | | 56(82.4) |  |
|  | Yes | 38(16.7) | | 26(16.3) | | 12(17.6) |  |
| Hypertensive |  |  | |  | |  | 0.291 |
|  | No | 184(80.7) | | 132(82.5) | | 52(76.5) |  |
|  | Yes | 44(19.3) | | 28(17.5) | | 16(23.5) |  |
| Diabetes |  |  | |  | |  | 0.948 |
|  | No | 195(85.5) | | 137(85.6) | | 58(85.3) |  |
|  | Yes | 33(14.5) | | 23(14.4) | | 10(14.7) |  |
| Liver Cirrhosis |  |  | |  | |  | 0.416 |
|  | No | 110(48.2) | | 80(50.0) | | 30(44.1) |  |
|  | Yes | 118(51.8) | | 80(50.0) | | 38(55.9) |  |
| HBsAg |  |  | |  | |  | 0.413 |
|  | No | 76(33.3) | | 56(35.0) | | 20(29.4) |  |
|  | Yes | 152(66.7) | | 104(65.0) | | 48(70.6) |  |
| AFP(ng/mL) |  |  | |  | |  | 0.278 |
|  | ≤400 | 163(71.5) | | 111(69.4) | | 52(76.5) |  |
|  | >400 | 65(28.5) | | 49(30.6) | | 16(23.5) |  |
| CEA(ng/mL) |  |  | |  | |  | 0.750 |
|  | ≤5 | 197(86.4) | | 138(86.9) | | 58(85.3) |  |
|  | >5 | 31(13.6) | | 21(13.1) | | 10(14.7) |  |
| ALB(g/L) |  |  | |  | |  | 0.566 |
|  | ≤35 | 45(19.7) | | 30(18.8) | | 15(22.1) |  |
|  | >35 | 183(80.3) | | 130(81.2) | | 53(77.9) |  |
| TBIL (umol/L) |  |  | |  | |  | 0.343 |
|  | ≤34.2 | 197(96.4) | | 136(85.0) | | 61(89.7) |  |
|  | >34.2 | 31(13.6) | | 24(15.0) | | 7(10.3) |  |
| ALP(U/L) |  |  | |  | |  | 0.247 |
|  | ≤135 | 163(71.5) | | 118(73.8) | | 45(66.2) |  |
|  | >34 | 65(28.5) | | 42(26.3) | | 23(33.8) |  |
| ALT (u/L) |  |  | |  | |  | 0.233 |
|  | ≤40 | 131(57.5) | | 96(60.0) | | 35(51.5) |  |
|  | >40 | 97(42.5) | | 64(40.0) | | 33(48.5) |  |
| AST (u/L) |  |  | |  | |  | 0.767 |
|  | ≤35 | 104(45.6) | | 74(46.3) | | 30(44.1) |  |
|  | >35 | 124(54.4) | | 86(53.8) | | 38(55.9) |  |
| HB(g/L) |  |  | |  | |  | 0.306 |
|  | ≤120 | 32(14.0) | | 20(12.5) | | 12(17.6) |  |
|  | >120 | 196(86) | | 140(87.5) | | 56(82.4) |  |
| PLT (10^9^/L) |  |  | |  | |  | 0.598 |
|  | ≤100 | 59(25.9) | | 43(26.9) | | 16(23.5) |  |
|  | >100 | 169(74.1) | | 117(73.1) | | 52(76.5) |  |
| PT(s) |  |  | |  | |  | 0.646 |
|  | ≤14 | 126(55.3) | | 90(56.3) | | 36(52.9) |  |
|  | >14 | 102(44.7) | | 70(43.8) | | 32(47.1) |  |
| INR |  |  | |  | |  | 0.990 |
|  | ≤1.3 | 218(95.6) | | 153(95.6) | | 65(95.6) |  |
|  | >1.3 | 10(4.4) | | 7(4.4) | | 3(4.4) |  |
| ALBI |  |  | |  | |  | 0.895 |
|  | ≤ -2.6 | 116(50.9) | | 83(51.9) | | 33(48.5) |  |
|  | -2.6- -2.39 | 102(44.7) | | 70(43.8) | | 32(47.1) |  |
|  | > -2.39 | 10(4.4) | | 7(4.4) | | 3(4.4) |  |
| PALBI |  |  | |  | |  | 0.936 |
|  | ≤-2.27 | 150(65.8) | | 105(65.6) | | 45(66.2) |  |
|  | >-2.27 | 78(34.2) | | 55(34.4) | | 23(33.8) |  |
| APRI |  |  | |  | |  | 0.174 |
|  | ≤0.5 | 71(31.3) | | 52(32.5) | | 19(27.9) |  |
|  | 0.5-1.5 | 103(45.2) | | 75(46.9) | | 28(41.2) |  |
|  | 1.5-2.5 | 32(14.0) | | 22(13.8) | | 10(14.7) |  |
|  | >2.5 | 22(9.6) | | 11(6.9) | | 11(16.2) |  |
| ANRI |  |  | |  | |  | 0.070 |
|  | ≤30 | 190(83.3) | | 138(86.2) | | 52(76.5) |  |
|  | >30 | 38(16.7) | | 22(13.8) | | 16(23.5) |  |
| PLR |  |  | |  | |  | 0.897 |
|  | ≤100 | 92(40.4) | | 65(40.6) | | 27(39.7) |  |
|  | >100 | 136(59.6) | | 95(59.4) | | 41(60.3) |  |
| FIB4 |  |  | |  | |  | 0.714 |
|  | ≤1.30 | 100(43.9) | | 71(44.4) | | 29(42.6) |  |
|  | 1.30-2.67 | 69(30.3) | | 50(31.3) | | 19(27.9) |  |
|  | >2.67 | 59(25.9) | | 39(24.4) | | 20(29.4) |  |
| Intraoperative blood loss (ml) |  | 451.18±580.846 | | 475.23±597.18 | | 414.71±542.35 | 0.473 |
| Number of tumors |  | 1.11±0.322 | | 1.10±0.321 | | 1.12±0.325 | 0.706 |
| Ascites |  |  | |  | |  | 0.154 |
|  | No | 181(79.4) | | 121(81.9) | | 50(73.5) |  |
|  | Yes | 47(20.6) | | 29(18.1) | | 18(26.5) |  |
| Tumor diameter(cm) |  |  | |  | |  | 0.542 |
|  | ≤5 | 117(51.3) | | 80(50.0) | | 37(54.4) |  |
|  | >5 | 111(48.7) | | 80(50.0) | | 31(45.6) |  |
| Tumor differentiation type |  |  | |  | |  | 0.416 |
|  | Highly - moderately | 159(69.7) | | 109(68.1) | | 50(73.5) |  |
|  | Poorly | 69(30.3) | | 51(31.9) | | 51(31.9) |  |
| Tumor thrombus |  |  | |  | |  | 0.390 |
|  | No | 176(77.2) | | 126(78.8) | | 50(73.5) |  |
|  | Yes | 52(22.8) | | 34(21.2) | | 18(26.5) |  |

Note: BMI, body mass index ;AFP, alpha-fetoprotein; CEA, Carcinoembryonic antigen; ALB, albumin; TBIL, total bilirubin; ALP, Alkaline phosphatase ; AST, aspartate transaminase; ALT, alanine transaminase; GGT, glutamyltranspeptidase; HB , hemoglobin; PLT, platelet count; PT, prothrombin time; INR, International normalized ratio; ALBI, albumin bilirubin score; PALBI, Platelet - Albumin - Bilirubin Index, APRI, aspartate transaminase to Platelet Ratio ; ANRI, aspartate aminotransferase-neutrophil ratio; PLR, platelet-to-lymphocyte ratio; FIB-4, fibrosis index;HBsAg, hepatitis B surface antigen;
